# Enhanced smartcard-based password-authenticated key agreement using extended chaotic maps

**DOI:** 10.1371/journal.pone.0181744

**Published:** 2017-07-31

**Authors:** Tian-Fu Lee, Chia-Hung Hsiao, Shi-Han Hwang, Tsung-Hung Lin

**Affiliations:** 1 Department of Medical Informatics, Tzu Chi University, Hualien, Taiwan, ROC; 2 Department of Medical Informatics, Institute of Medical Sciences, Tzu Chi University, Hualien, Taiwan, ROC; 3 Department of Computer Science and Information Engineering, National Chin-Yi University of Technology, Taichung, Taiwan, ROC; King Saud University, SAUDI ARABIA

## Abstract

A smartcard based password-authenticated key agreement scheme enables a legal user to log in to a remote authentication server and access remote services through public networks using a weak password and a smart card. Lin recently presented an improved chaotic maps-based password-authenticated key agreement scheme that used smartcards to eliminate the weaknesses of the scheme of Guo and Chang, which does not provide strong user anonymity and violates session key security. However, the improved scheme of Lin does not exhibit the freshness property and the validity of messages so it still fails to withstand denial-of-service and privileged-insider attacks. Additionally, a single malicious participant can predetermine the session key such that the improved scheme does not exhibit the contributory property of key agreements. This investigation discusses these weaknesses and proposes an enhanced smartcard-based password-authenticated key agreement scheme that utilizes extended chaotic maps. The session security of this enhanced scheme is based on the extended chaotic map-based Diffie-Hellman problem, and is proven in the real-or-random and the sequence of games models. Moreover, the enhanced scheme ensures the freshness of communicating messages by appending timestamps, and thereby avoids the weaknesses in previous schemes.

## Introduction

Smartcard-based password-authenticated key agreement supports a communicating platform that enables legitimate users to log in to, and access, systems conveniently and securely over an open network. In a smartcard-based password-authenticated key agreement system, users register their identities and passwords with a trusted server. The trusted server is then responsible for generating authentication information and secrets of users and providing smartcards to legitimate users over a secure and authenticated channel. Finally, legitimate users conveniently and securely log in and enjoy remote services using their weak passwords and smartcards [1–8].

Recently, Chen et al. [9] developed a smartcard-based password authentication scheme based on the Discrete Logarithm problem and claimed that their scheme can withstand potential attacks. However, Jiang et al. [10] stated that their scheme is insecure against offline password guessing attacks, and presented an improved authentication scheme based on the Diffie-Hellman problem to solve the security flaw of the scheme of Chen et al. and to keep efficiency. In 2013, Wen [11] designed an enhanced user authentication scheme based on the quadratic residue problem [12, 13] to overcome the weaknesses of previous schemes [14, 7]. However, Islam et al. [15] pointed out the security weaknesses of Wen’s scheme, and showed that their scheme cannot resist some possible attacks, including impersonation and privileged-insider attacks. Islam et al. also presented a new user authentication scheme based on the quadratic residue problem for the application of integrated EPR information system. Additionally, Li [16] developed a two-factor authentication scheme with user anonymity based on elliptic curve cryptography. But, Wang et al. [17] showed that his scheme may suffer from smart-card loss and de-synchronization attacks, and provided a better understanding of the underlying evaluation metric for anonymous two-factor schemes. These schemes [9–16] are developed by using public-key cryptosystem to have higher security. Nevertheless, time-consuming modular exponential computations are required so that these schemes are inefficient in computation.

Since cryptography that uses chaotic maps was demonstrated to exhibit the semi-group property and cryptosystems that use chaotic map operations were shown to be more efficient than cryptosystems that use modular exponential computations and scalar multiplications on the elliptic curve [18–20], many chaotic map-based authentication approaches [21–29] have been developed. However, in 2005, Bergamo et al. [20] showed the security weakness of public-key cryptosystems that are based on Chebyshev polynomials, and that therefore some authentication schemes have security limitations and lack the contributory property of key agreements. In 2008, Zhang [30] enhanced the Chebyshev polynomials to eliminate this security weakness. Zhang also demonstrated that the enhanced Chebyshev polynomials support the semi-group property and the commutivity under composition on interval (−∞,+∞). Additionally, extended Chebyshev chaotic maps are utilized in solving the extended chaotic map-based discrete logarithm and Diffie-Hellman problems [30–32]. In 2013, Guo and Change [33] were the first to present a novel chaotic map-based password-authenticated key agreement scheme using smartcards to increase efficiency. In 2014, Lin [34] developed a mobile user authentication scheme using dynamic identity and chaotic map, and declared that their scheme offers mutual authentication, session key security and user anonymity, and resilience against possible attacks. Later, Islam et al. [35] stated that Lin’s scheme had some design flaws and limitations, and cannot resist user impersonation attack. Islam et al. also presented a provably secure scheme using extended chaotic map to solve the weaknesses of Lin’s scheme. Additionally, Islam [36] in 2014 proposed a dynamic identity-based three-factor scheme using extended chaotic maps three-factor authentication to offer more security properties. However, Jiang et al. [37] pointed out the processing flaws of Islam’s scheme, and showed that his scheme is also vulnerable to some potential attacks. To solve these limitations, Jiang et al. also presented a more secure robust three-factor authentication scheme. Subsequently, Hao et al. [38], Lee [39] and Lin [40] noted that the scheme developed by Guo and Chang had weaknesses that included an inability to ensure strong user anonymity, inefficiency in hiding double secrets, and violation of both the session key security and the contributory property of key agreements. Lin [41] also proposed an improved scheme to eliminate the weaknesses in the scheme of Guo and Chang. However, Lin’s scheme also failed to withstand some attacks and to meet all security requirements. In the password change phase of that scheme, the server does not confirm the freshness of the messages from the users, and the smartcard does not verify the updated data from the server, so the scheme fails efficiently to protect against replay and denial of service attacks. Additionally, in the authenticated key exchange phase, a malicious server can control the value of a session key by the method that was introduced by Bergamo et al. [20] so Lin’s scheme also the fails to provide the contributory property of key agreements. Moreover, in that scheme, every legitimate user can derive session key that is shared between another user and the server by the method of Bergamo et al. [20]. A malicious user can even forge validate request messages and to impersonate other users, so Lin’s scheme fails to withstand privileged-insider attacks.

To address the weaknesses of Lin’s scheme, this work develops a more secure and efficient smartcard-based password-authenticated key agreement scheme that is based on the schemes of both Guo and Chang [33] and Lin [40]. The enhanced scheme constructs the session key using extended chaotic maps, and so the session key of security is based on the extended chaotic map-based Diffie-Hellman problem. The enhanced scheme eliminates the security weakness that was identified by Bergamo et al.; ensures the contributory property of key agreements, and withstands attacks by privileged insiders. Moreover, in the password change phase of the enhanced scheme, the messages are guaranteed to exhibit freshness property owing to the appending of timestamps, so the enhanced scheme withstands replay and denial-of-service attacks. Therefore, the proposed scheme does not have any of the weaknesses of previous schemes.

The remainder of this article is organized as follows. Section 2 describes the notation and the definitions used in this paper. Section 3 reviews the authenticated key agreement scheme of Lin and elucidates its weaknesses. Section 4 presents the enhanced smartcard-based password-authenticated key agreement that uses extended chaotic maps. Section 5 analyzes the security and performance of the enhanced scheme. Finally, Section 6 draws conclusions.

## Preliminaries

This section presents the notation and the definitions that are used herein this work.

### Notation

The followings detail the notation that is utilized herein.

*U*: The user
*ID*: The identity of *U*
*PW*: The password of *U*
*S*: The remote server, which *U* is registered in
*T*_1_: The user’s time stamp
*T*_2_: The server’s time stamp
Δ*T*: The time threshold
*E*_*k*_(·)/*D*_*k*_(·): A secure symmetric en/decryption algorithm with the secret key *k*
*λ*: The session key generated between *U* and *S*
*l*: The secure parameter size
*h*(·): A one-way hash function and *h*:{0,1}*→{0,1}^*l*^
*H*(.): A one-way hash function and *H*:[−1,1]→{0,1}^*l*^
*A*→*B* : *M*: *A* sends message *M* to *B* through a common channel.
*M*_1_||*M*_2_: Message *M*_1_ concatenates to message *M*_2_.

### Definition

#### Session key security (AKE security)

This definition defines that an adversary A fails to effectively distinguish between two messages from a challenger C. One message is encrypted with the real session key *λ* and the other one is encrypted with a random string *λ*’ via an unbiased coin *c*. A selects one message and sends it to C. Then C flips an unbiased coin *c* ∈ {0,1} and decides to return the message encrypted with *λ* if *c* = 1 or encrypted with *λ*′ if *c* = 0. A intends to correctly guess the value of the hidden bit. The advantage that an adversary A violates the indistinguishability of a scheme **P** is denoted as $Adv_{P}^{ake}$(A). The scheme **P** is AKE-secure if $Adv_{P}^{ake}$(A) is negligible. [41–44]

#### Chebyshev chaotic maps

The Chebyshev polynomial *T*_*n*_(*x*) is a polynomial in *x* of degree *n* and is defined by the following relation:

$$T_{n}(x)=\text{cos}n\theta ,\text{ where }x=\text{cos}\theta .$$

The recurrence relation of *T*_*n*_(*x*) is defined as:

$$T_{n}(x)=2xT_{n-1}(x)-T_{n-2}(x),$$

for any *n* ≥ 2, with *T*_0_(*x*) = 1 and *T*_1_(*x*) = *x*.

The Chebyshev polynomial satisfies the semi-group property and satisfies:

$$T_{r}(T_{s}(x))=T_{sr}(x)=T_{s}(T_{r}(x)),$$

for *s*,*r* ∈ *Z*^+^.

The Chebyshev polynomial satisfies chaotic property: When *n >* 1, Chebyshev polynomial map *T*_*n*_: [−1,1]→[−1,1] of degree *n* is a chaotic map with its invariant density

$$f^{*}(x)=1/\left(\pi \sqrt{1-x^{2}}\right),$$

for Lyaounov exponent ln *n* > 0.[29–32]

Zhang [30] in 2008 enhanced the Chebyshev polynomials for avoiding the security weakness showed by Bergamo et al. [20] in 2005, and also proved that the enhanced Chebyshev polynomials still satisfy the semi-group property and the commutative under composition on interval (−∞,+∞). That is,

$$T_{n}(x)\equiv (2xT_{n-1}(x)-T_{n-2}(x))\text{mod}p,$$

where *n* ≥ 2, *x* ∈ (−∞,+∞) and *p* is a large prime number. Then,

$$T_{r}(T_{s}(x))\equiv T_{rs}(x)\equiv T_{s}(T_{r}(x))\text{mod}p$$

holds.

The enhanced Chebyshev chaotic maps also exhibit the Discrete Logarithm and Diffie-Hellman problems [30–32], which are described as follows.

#### Extended chaotic map-based discrete logarithm problem (DLP)

Given *x*, *y* and *p*, finding the integer *r* satisfying *y* = *T*_*r*_(*x*) mod *p* is computationally infeasible. The advantage that an adversary solves the extended chaotic map-based DLP is denoted as *Adv*^*dlp*^, and thus is negligible.

#### Extended chaotic map-based computational Diffie-Hellman problem (CDHP)

Given *T*_*r*_(*x*), *T*_*s*_(*x*), *T*(·), *x* and *p*, where *r*, *s* ≥ 2, *x* ∈ (−∞,+∞) and *p* is a large prime number, calculating

$$T_{rs}(x)\equiv T_{r}(T_{s}(x))\equiv T_{s}(T_{r}(x))\text{mod}p$$

is computationally infeasible. The advantage that an adversary solves the extended chaotic map-based CDHP is denoted as *Adv*^*cdh*^, and thus is negligible.

#### Extended chaotic map-based decisional Diffie-Hellman problem (DDHP)

Given *T*_*r*_(*x*), *T*_*s*_(*x*), *T*_*z*_(*x*), *T*(·), *x* and *p*, deciding whether

$$T_{rs}(x)\equiv T_{z}(x)\text{mod}p$$

holds or not is computationally infeasible. The advantage that an adversary solves the extended chaotic map-based DDHP is denoted as *Adv*^*ddh*^, and thus is negligible.

## The authenticated key agreement scheme of Lin and its limitations

### The authenticated key agreement scheme of Lin

Lin [40] recently presented an improved chaotic maps-based password authenticated key agreement scheme using smartcards. The four phases of the improved scheme are system initialization, user registration, authenticated key exchange and password change phases, which are discussed further below.

#### System initialization phase

The remote server *S* setups the system’s parameters by performing the following steps:

1. *S* generates a random number *r* as the private key and a random number *x* ∈ [−1,+1].
2. *S* chooses a master key *s*, a secure symmetric en/decryption algorithm *E*_*k*_(·)/*D*_*k*_(·) and a one-way hash function *h*(·).

#### Registration phase

A user *U* registers his/her identity and password by performing the following steps.

1. *U* chooses his identity *ID*, password *PW* and a random number *t* and sends *ID* and *H* = *h*(*PW* ∥ *t*) to *S* via a secure channel.
2. *S* verifies *ID* and computes *R* = *E*_*s*_(*ID* ∥ *H*) and *D* = *H* ⊕ (*x* ∥ *T*_*r*_(*x*)) by using its master key *s*.
3. *S* stores (*R*,*h*(·),*E*_*k*_(·),*D*) into a smartcard *SC*, and issue *SC* to *U* through a secure channel.
4. *U* inserts *t* into it and finishes the registration.

#### Authenticated key exchange phase

**Fig 1:**
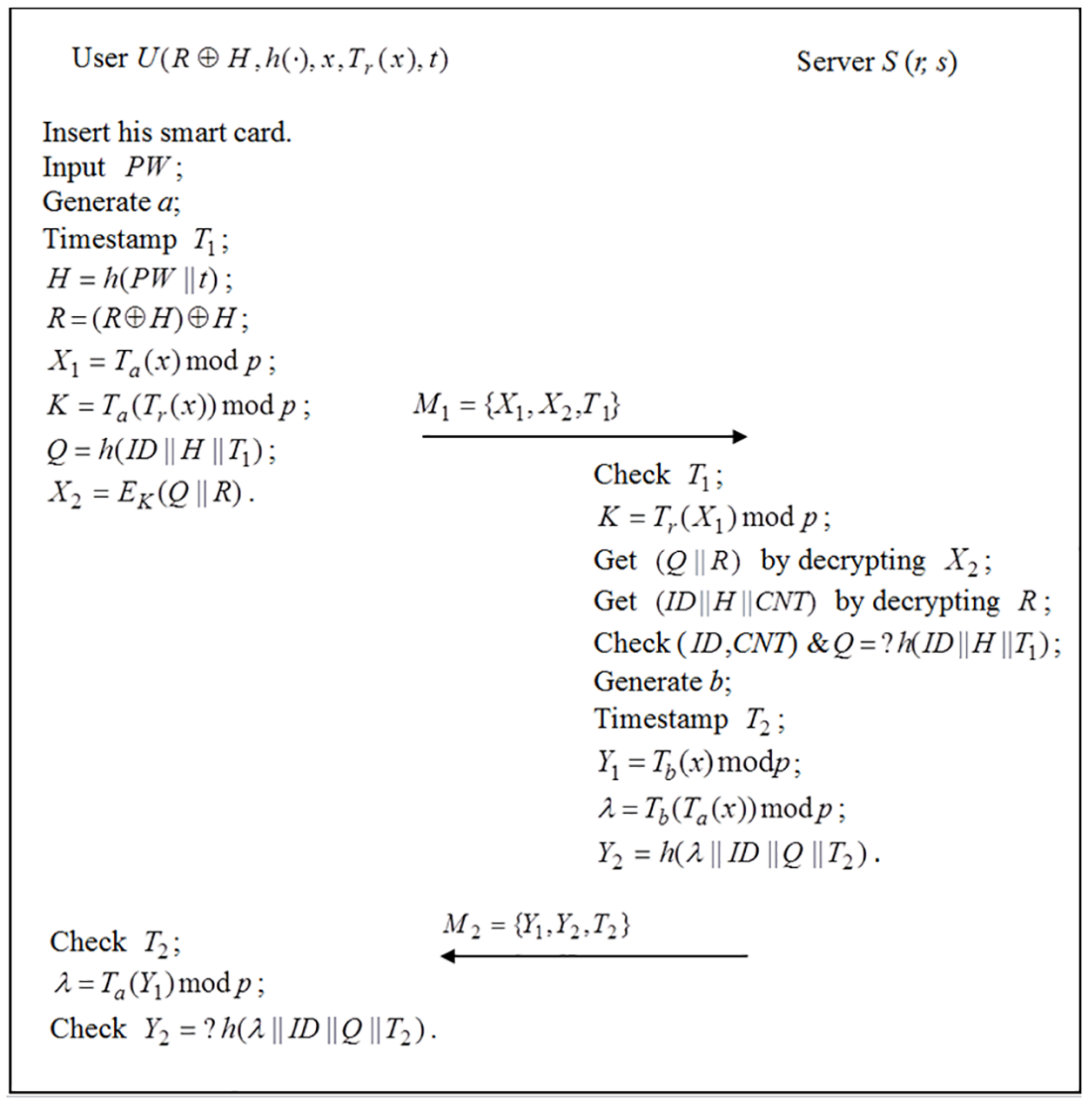
The authenticated key exchange phase of the enhanced scheme.

In this phase, as shown in Fig 1, *U* and *S* authenticate each other by performing the following steps.

1. *U* inserts his *SC* into a card reader and inputs *PW*. Then *SC* generates a random number *j*, computes *T*_*j*_(*x*), (*x* ∥ *T*_*r*_(*x*)) = *h*(*PW* ∥ *t*) ⊕ *D*, *v* = *T*_*j*_(*T*_*r*_(*x*)), *Q* = *h*(*ID* ∥ *H*), *E*_*v*_(*Q* ∥ *R* ∥ *T*_1_), where *T*_1_ is the current timestamp, and sends *M*_1_ = {*T*_*j*_(*x*),*E*_*v*_(*Q* ∥ *R* ∥ *T*_1_)} to *S*.
2. On receiving *M*_1_, *S* computes *v* = *T*_*r*_(*T*_*j*_(*x*)), obtains (*Q* ∥ *R* ∥ *T*_1_) by decrypting *E*_*v*_(*Q* ∥ *R* ∥ *T*_1_) with *v*, and checks *T*_1_. If unsuccessful, *S* rejects this service request. Otherwise, *S* obtains (*ID*′ ∥ *H*′) by decrypting *R* with its master key *s* and checks whether *Q*′ = ?*h*(*ID*′ ∥ *H*′). If unsuccessful, *S* rejects this service request. Otherwise, *S* generates a random number *j*′, and computes *T*_*j*′_(*x*) and *E*_*v*_(*T*_*j*′_(*x*) ∥ *h*(*ID* ∥ *T*_2_) ∥ *T*_2_), where *T*_2_ is the current timestamp, and sends *E*_*v*_(*T*_*j*′_(*x*) ∥ *h*(*ID* ∥ *T*_2_) ∥ *T*_2_) to *SC*.
3. On receiving *E*_*v*_(*T*_*j*′_(*x*) ∥ *h*(*ID* ∥ *T*_2_) ∥ *T*_2_), *SC* obtains (*T*_*j*′_(*x*) ∥ *h*′(*ID* ∥ *T*_2_) ∥ *T*_2_) by decrypting *E*_*v*_(*T*_*j*′_(*x*) ∥ *h*(*ID* ∥ *T*_2_) ∥ *T*_2_) with *v* and checks *T*_2_. If unsuccessful, the *SC* aborts this service request. Otherwise, *SC* checks whether *h*′(*ID* ∥ *T*_2_) = ?*h*(*ID* ∥ *T*_2_). If unsuccessful, *SC* aborts this service request. Finally, both *U* and *S* share a common session key *λ* = *T*_*j*′_(*T*_*j*_(*x*)) = *T*_*j*_(*T*_*j*′_(*x*)).

#### Password change phase

A legal user *U* inserts his *SC* into a card reader and inputs the old password *PW* and a new password PW* and changes his/her password by performing the following steps.

1. The *SC* generates a random number *i*, computes *H*′ = *h*(*PW* ∥ *t*), (*x* ∥ *T*_*r*_(*x*)) = *h*(*PW* ∥ *t*) ⊕ *D*, *η* = *T*_*i*_(*T*_*r*_(*x*)), H* = *h*(PW* ∥ *t*), and sends (*T*_*i*_(*x*),*E*_*η*_(*H*′ ∥ H* ∥ *R*)) to *S*.
2. On receiving (*T*_*i*_(*x*),*E*_*η*_(*H*′ ∥ H* ∥ *R*)), *S* computes *v* = *T*_*r*_(*T*_*i*_(*x*)), obtains (H* ∥ *Q* ∥ *R*) by decrypting *E*_*η*_(*H*′ ∥ H* ∥ *R*) with *v* and obtains (*ID* ∥ *H*) by decrypting *R* with *s*, respectively. Then *S* checks whether *H*′ = ?*H* holds or not. If successful, *S* computes R* = *E*_*s*_(*ID* ∥ H*) and sends R* to *SC*.
3. After receiving R*, *SC* updates *R* as R*.

### Weaknesses in the authenticated key agreement scheme of Lin

This subsection elucidates the weaknesses of the improved scheme of Lin, which suffers from denial-of-service attacks and privileged-insider attacks, and violation of the contributory property of key agreements.

#### Suffering from denial-of-service attacks

In the password change phase, the smartcard does not validate the updated data *R* so an attacker can easily perform a denial-of-service by the following steps.

1. On receiving message (*T*_*i*_(*x*),*E*_*η*_(*H*′,H*,*R*)) from a user, the server computes *η* = *T*_*r*_(*T*_*i*_(*x*)), decrypts *E*_*η*_(*H*′,H*,*R*) and *R* = *E*_*s*_(*ID* ∥ *H*) using *η* and the server’s master key *s*, respectively, and then checks whether *H*′ = ?*H*.
2. If *H*′ = *H*, then *S* returns R* = *E*_*s*_(*ID* ∥ H*) to the smart card. At this time, an attacker intercepts R* and replaces it with a nonce $\hat{R}$.
3. On receiving message $\hat{R}$, the smartcard does not verify it but updates *R* as $\hat{R}$. Thereafter, when the user attempts to implement the steps of the authenticated key exchange phase or the password change phase, the failed request message $\left(T_{j}(x),E_{v}(Q,\hat{R},T_{1})\right)$ or $\left(T_{i}(x),E_{\eta }(H^{\prime },H^{*},\hat{R})\right)$ will be detected by the server because the user does not have the correct *R*. Thereafter, the server always rejects the service requests made by the user. Therefore, the scheme of Lin is insecure against denial-of-service attacks.

Moreover, in the password change phase, the server does not verify the freshness of messages from the users so an attacker can exhaust computational resources in the server by replaying previous request messages. Possible scenarios are as follows.

1. After the user sends the message (*T*_*i*_(*x*), *E*_*η*_(*H*′,H*,*R*)) to the server, an attacker can copy it and successively re-send it to the server.
2. Upon receiving each message (*T*_*i*_(*x*), *E*_*η*_(*H*′,H*,*R*)) from the attacker, the server computes *η* = *T*_*r*_(*T*_*i*_(*x*)), decrypts *E*_*η*_(*H*′,H*,*R*) and *R* = *E*_*s*_(*ID* ∥ *H*), and successfully checks whether *H*′ = *H*. Then, the server computes and returns R* = *E*_*s*_(*ID* ∥ H*). The server may exhaust computational resources and cannot efficiently prevent denial-of-service attacks since the server does not verify the freshness of these request messages.

#### Suffering from privileged insider attacks

In Lin’s authentication scheme, every legitimate user can derive (*x* ∥ *T*_*r*_(*x*)) from his/her smartcard. A malicious user U* still can derive the session key that is shared between another user *U* and the server using the method that was introduced by Bergamo et al. [20]. The details are as follows.

1. After the user *U* sends out the message (*T*_*j*_(*x*),*E*_*v*_(*Q*,*R*,*T*_1_)), U* receives *T*_*j*_(*x*). By the method of Bergamo *et al*., U* possesses *x*, *T*(·), *T*_*r*_(*x*) and *T*_*j*_(*x*), and so can compute an integer solution j* that satisfies the equation $T_{j^{*}}(x)=T_{j}(x)$:
  $$j^{*}=\frac{\text{arccos}(T_{j}(x))+2k\pi }{\text{arccos}(x)}|k\in Z.$$
2. U* can compute the secret key $v=T_{j^{*}}(T_{r}(x))$ since $T_{j^{*}}(T_{r}(x))=T_{r}(T_{j^{*}}(x))=T_{r}(T_{j}(x))=v$. Then, U* receives *R* by decrypting *E*_*v*_(*Q*,*R*,*T*_1_) using *v*, and can determine whether two request messages came from the same user.
3. After the server returns the message *E*_*v*_(*T*_*j*′_(*x*), *h*(*ID* ∥ *T*_2_), *T*_2_), U* receives *T*_*j*′_(*x*) and so can compute the session key $\lambda =T_{j^{*}}(T_{j^{\prime }}(x))$ since $T_{j^{*}}(T_{j^{\prime }}(x))=T_{j^{\prime }}(T_{j^{*}}(x))=T_{j^{\prime }}(T_{j}(x))=\lambda$. Furthermore, U* can impersonate another user *U* by forging a request message $\left(T_{j^{*}}(x),E_{v^{*}}(Q,R,{T^{\prime }}_{1})\right)$, where ${T^{\prime }}_{1}$ is an acceptable timestamp and $v^{*}=T_{j^{*}}(T_{r}(x))$, since U* has *x*, *T*_*r*_(*x*), *Q* and *R*.

Therefore, Lin’s authentication scheme fails to withstand privileged insider attacks since every legitimate user has *x* and *T*_*r*_(*x*), and can derive users’ hidden information concerning *Q* and *R*.

#### Lack of the contributory property of key agreements

In the authenticated key exchange phase of the authenticated key agreement scheme of Lin, the malicious server alone can control the value of the session key using the method proposed by Bergamo et al. [20]. The details are as follows.

1. Upon receiving the message from a user, the malicious server *S* receives *T*_*j*_(*x*) and computes an integer solution j* to the equation $T_{j^{*}}(x)=T_{j}(x)$:
  $$j^{*}=\frac{\text{arccos}(T_{j}(x))+2k\pi }{\text{arccos}(x)}|k\in Z.$$
2. *S* uses a predetermined value *λ*_0_ to find an integer j′, using
  $$j^{\prime }=\frac{\text{arccos}(\lambda_{0})+2k\pi }{j^{*}\cdot \text{arccos}(x)}|k\in Z;$$
  calculates *E*_*v*_(*T*_*j*′_(*x*) ∥ *h*(*ID* ∥ *T*_2_) ∥ *T*_2_), and sends it to the smart card.
3. Upon receiving the message from *S*, the smartcard receives (*T*_*j*′_(*x*) ∥ *h*(*ID* ∥ *T*_2_) ∥ *T*_2_) by decrypting *E*_*v*_(*T*_*j*′_(*x*) ∥ *h*(*ID* ∥ *T*_2_) ∥ *T*_2_); it then computes *T*_*j*_(*T*_*j*′_(*x*)) as the session key. Therefore, *U* obtains the session key *λ*_0_ because $T_{j}(T_{j^{\prime }}(x))=T_{j^{\prime }}(T_{j}(x))=T_{j^{\prime }}(T_{j^{*}}(x))=\lambda_{0}$.

Therefore, Lin’s scheme does not support the contributory property of key agreements because the malicious server can control the value of the session key.

## Enhanced smartcard-based password-authenticated key agreement scheme

This section elucidates the enhanced smartcard-based password-authenticated key agreement scheme that uses extended chaotic maps. The session key security of the enhanced scheme is based on the extended chaotic map-based Diffie-Hellman problem so one malicious participant cannot alone predetermine the value of the session key. Additionally, malicious users cannot derive the mutually session key that is shared between another user and the server, and they cannot forge validate request messages or impersonate other users. Thus, the enhanced scheme withstands privileged insider attacks. Moreover, in the password change phase of the enhanced scheme, the appending of timestamps guarantees the freshness of messages that are sent from users, and the smartcard can validate the updated data from the server, so the enhanced scheme withstands replay and denial-of-service attacks.

The enhanced scheme consists of five phases, which are system initialization, user registration, authenticated key exchange, password change, and smartcard revocation phases. The system initialization phase is similar to those of Lin’s scheme, except that it uses enhanced Chebyshev chaotic maps and the parameter *x* on interval (−∞,+∞), requires a large prime number *p* for the modular arithmetic, and maintains a smartcard revocation table in the system initialization phases. The registration, authenticated key exchange, password change and smartcard revocation phases are described further below.

### Registration phase

A user *U* registers his/her identity and password to be a legal user by performing the following steps.

1. *U* chooses his identity *ID*, password *PW* and a random number *t* and sends *ID* and *H* = *h*(*PW* ∥ *t*) to *S* via a secure channel.
2. *S* verifies *ID* and computes *R* = *E*_*s*_(*ID* ∥ *H* ∥ *CNT*) by using its master key *s*, where *CNT* = 0 and indicates the revocation times.
3. *S* stores (*R* ⊕ *H*,*h*(·),*E*_*k*_(·),*x*,*T*_*r*_(*x*)) into a smartcard *SC*, issue the *SC* to *U* through a secure channel.
4. After receiving *SC*, *U* inserts *t* into it and finishes the registration.

### Authenticated key exchange phase

In this phase, as shown in Fig 1, the user *U* and the server *S* authenticate each other and negotiate a common session key by performing the following steps.

1. *U* inserts his *SC*, inputs *PW*, computes *H* = *h*(*PW* ∥ *t*) and *R* = (*R* ⊕ *H)* ⊕ *H*, generates a random number *a*, calculates *X*_1_ = *T*_*a*_(*x*)mod *p*, *K* = *T*_*a*_(*T*_*r*_(*x*))mod *p*, *Q* = *h*(*ID* ∥ *H* ∥ *T*_1_), *X*_2_ = *E*_*K*_(*Q* ∥ *R*), where *T*_1_ is the current timestamp, and sends *M*_1_ = {*X*_1_,*X*_2_,*T*_1_} to *S*.
2. On receiving *M*_1_, *S* checks whether *T*′−*T*_1_ ≤ Δ*T* holds or not, where *T*′ is the current timestamp. If unsuccessful, *S* aborts this service request; Otherwise *S* computes *K* = *T*_*r*_(*X*_*1*_) mod *p*, obtains (*Q* ∥ *R*) by decrypting *X*_2_with *K* and obtains (*ID* ∥ *H* ∥ *CNT*) by decrypting with *s*, respectively. Then *S* checks whether (*ID*, *CNT*) is recorded in its revocation table or not and verifies *Q* = ?*h*(*ID* ∥ *H* ∥ *T*_1_). If unsuccessful, *S* still rejects this service request; Otherwise *S* generates random numbers *b*, computes *Y*_*1*_ = *T*_*b*_(*x*)mod*p*, the session key *λ* = *T*_*b*_(*T*_*a*_(*x*))mod *p* and *Y*_2_ = *h*(*λ* ∥ *ID* ∥ *Q* ∥ *T*_2_), where *T*_1_ is the current timestamp, and sends *M*_2_ = {*Y*_1_,*Y*_2_,*T*_2_} to *U*.
3. On receiving *M*_2_, *U* checks whether *T*″−*T*_2_ ≤ Δ*T* holds or not, where *T*″ is the current timestamp. If unsuccessful, *U* omits this service request; Otherwise *U* computes the session key *λ* = *T*_*a*_(*Y*_1_)mod*p* and checks whether *Y*_2_ = ?*h*(*λ* ∥ *ID* ∥ *Q* ∥ *T*_2_) holds or not. If unsuccessful, *U* still omits this service request.

### Password change phase

**Fig 2:**
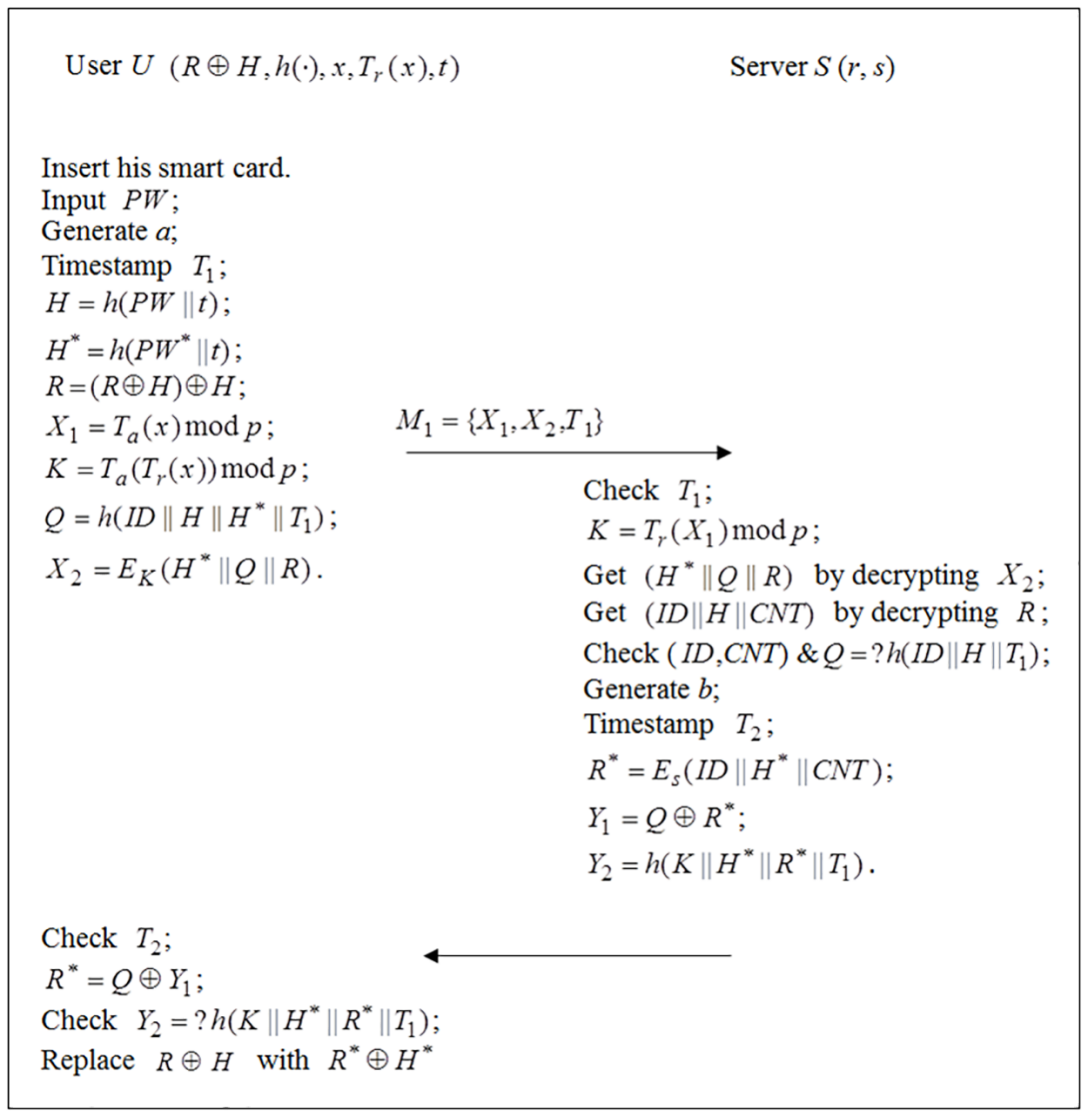
The password change phase of the enhanced scheme.

In this password change phase, as shown in Fig 2, a legal user inserts his/her smartcard *SC* and inputs the old password *PW* and a new password PW*, and then changes the password by performing the following steps.

1. *SC* computes *H* = *h*(*PW* ∥ *t*), H* = *h*(PW* ∥ *t*), generates a random number *a*, calculates *X*_1_ = *T*_*a*_(*x*) mod *p*, *K* = *T*_*a*_(*T*_*r*_(*x*)) mod *p*, *Q* = *h*(*ID* ∥ *H* ∥ H* ∥ *T*_*1*_), *R* = (*R* ⊕ *H*) ⊕ *H* and *X*_2_ = *E*_*K*_(H* ∥ *Q* ∥ *R*), where *T*_1_ is the current timestamp, and sends *M*_1_ = {*X*_1_,*X*_2_,*T*_1_} to the server.
2. On receiving *M*_1_, *S* checks whether *T*′−*T*_1_ ≤ Δ*T* holds or not, where *T*′ is the current timestamp. If unsuccessful, *S* aborts this service request; Otherwise *S* computes *K* = *T*_*r*_(*X*_*1*_) mod *p*, obtains (H* ∥ *Q* ∥ *R*) by decrypting *X*_2_ with *K* and obtains (*ID* ∥ *H* ∥ *CNT*) by decrypting with *s*, respectively. Then *S* checks whether (*ID*, *CNT*) is recorded in its revocation table or not and verifies *Q* = ?*h*(*ID* ∥ *H* ∥ *T*_1_). If successful, *S* computes R* = *E*_*s*_(*ID* ∥ H* ∥ *CNT*), *Y*_1_ = *Q* ⊕ R* and *Y*_2_ = *h*(*K* ∥ H* ∥ R* ∥*T*_1_), and sends *M*_2_ = {*Y*_1_,*Y*_2_} to the smartcard.
3. On receiving *M*_2_, *SC* computes R* = *Q* ⊕ *Y*_1_ and checks whether $Y_{2}=?h(K∥H^{*}∥R^{*}∥T_{1})$ holds or not. If successful, the smartcard replaces *R* ⊕ *H* with R* ⊕ H*.

### Smartcard revocation phase

This phase enables a legal user to revoke his/her old smartcard and to issue a new smartcard by performing the following steps.

1. *U* inputs his/her identity *ID*, password *PW*, selects a random number *t*_*new*_, computes *H*_*new*_ = *h*(*PW* ∥ *t*_*new*_), and sends {*ID*, *H*_*new*_, *Smartcard Revocation Request*} to *S* via a secure channel.
2. *S* searches (*ID*,*CNT*) in its revocation table, computes *CNT*_*new*_ = *CNT*+1 and *R*_*new*_ = *E*_*s*_(*ID* ∥ *H*_*new*_ ∥ *CNT*_*new*_) by using its master key *s*, and stores (*ID*,*CNT*_*new*_) in its revocation table.
3. *S* stores (*R*_*new*_,*h*(·),*E*_*k*_(·),*x*,*T*_*r*_(*x*)) into a smartcard, and issue the smartcard to *U* through a secure channel.
4. After receiving the smartcard, *U* inserts *t*_*new*_ into it and finishes the smartcard revocation processes.

## Security and performance analyses

### Security analysis

This subsection analyzes the security of the enhanced scheme, with reference to session key security, the contributory property of key agreements, and the withstanding of replay, denial-of-service and privileged-insider attacks.

Since the enhanced scheme is based on the schemes of Guo and Chang and Lin, the analyses of security requirements and the withstanding of possible attacks closely resemble those for the schemes of Guo and Chang and Lin, and so are not presented here.

#### Providing session key security (AKE security)

The following descriptions reveal that the enhanced scheme provides session key security by adopting the real-or-random (ROR) and the sequence of games (SOG) models [41–45].

The Difference Lemma [45] is used for the sequence of games and is described as follows:

Lemma 1 (Difference Lemma). Let *A*, *B* and *F* be events defined in some probability distribution, and suppose that A∧¬F⇔B∧¬F. Then

|Pr[A]−Pr[B]|≤Pr[F].

The following theorem shows that the proposed scheme has AKE security if the extended chaotic map-based DDHP holds.

Theorem 1. The probability that an adversary breaks the AKE security of the enhanced authenticated key agreement scheme *P* satisfies,

$$Adv_{P}^{ake}\leq 2\cdot Adv^{ddh}+\frac{2}{N}+\frac{1}{2^{l-1}},$$

where *Adv*^*ddh*^ is the advantage that an extended chaotic map-based *DDH* attacker can gain by solving the extended chaotic map-based *DDHP*, *N* is the size of password lists, and *l* is a secure parameter size.

Proof: Game ${\text{G}}_{i}^{ake}$ defines the probability of the event *E*_*i*_ that the adversary wins this game. The start game ${\text{G}}_{0}^{ake}$ is a real attack against the proposed scheme, and the final game ${\text{G}}_{\text{1}}^{ake}$ ends a negligible advantage gained by an attacked by breaking the AKE security of the enhanced scheme.

Game ${\text{G}}_{0}^{ake}$: This game corresponds to the real attack. By definition,

$$Adv_{P}^{ake}(\text{A})=|2\text{Pr}[E_{\text{0}}]-1|\text{.}$$ (1)

Game ${\text{G}}_{\text{1}}^{ake}$: This game considers password-guessing attacks. Each *X*_2_ = *E*_*K*_(*Q* ∥ *R*) is different, where *Q* = *h*(*ID* ∥ *H* ∥ *T*_1_), *H* = *h*(*PW* ∥ *t*) and *K* = *T*_*a*_(*T*_*r*_(*x*)) mod *p*, since *t* and *a* are random numbers selected by user *U*, and *T*_1_ is the timestamp. Thus, the adversary has no information for verifying his/her password guesses. This implies that the security against password attacks is measured by the probability that exists messages of the form *X*_2_ = *E*_*K*_(*Q* ∥ *R*) such that the guessing password is correct. Then, we have

$$|\text{Pr}[E_{\text{0}}]-\text{Pr}[E_{\text{1}}]|\leq \frac{1}{N}.$$ (2)

Game ${\text{G}}_{\text{2}}^{ake}$: This game transforms game **${\text{G}}_{\text{1}}^{ake}$** into game **${\text{G}}_{\text{2}}^{ake}$**, getting *Q* by choosing a random number, instead of computing a hash. Then, games ${\text{G}}_{\text{1}}^{ake}$ and ${\text{G}}_{\text{2}}^{ake}$ are undistinguishable except collisions of a hash function in ${\text{G}}_{\text{2}}^{ake}$. Thus, according to the birthday paradox [42] and Lemma 1, we have

$$|\text{Pr}[E_{\text{1}}]-\text{Pr}[E_{\text{2}}]|\leq \frac{1}{2^{l}}.$$ (3)

Game ${\text{G}}_{\text{3}}^{ake}$: This game is transformed from game **${\text{G}}_{\text{2}}^{ake}$** by using a triple (*X*,*Y*,*Z*) sample from a random distribution (*T*_*a*_(*x*)mod *p*,*T*_*b*_(*x*)mod *p*,*T*_*z*_(*x*)mod *p*), rather than an extended chaotic map-based DDH triple. ${\text{G}}_{\text{2}}^{ake}$ is therefore equivalent to ${\text{G}}_{\text{3}}^{ake}$, and

$$\text{Pr}[E_{\text{2}}]=\text{Pr}[E_{\text{3}}].$$ (4)

Let a challenger A_ddh_ attempt to violate the indistinguishability of the extended chaotic map-based DDHP, and let an adversary A_ake_ be created to violate the session key security. A_ddh_ returns the real key *λ* to A_ake_ if the flipping unbiased coin bit *c* = 1; otherwise, *c* = 0 and it returns a random string to A_ake_. Then A_ake_ outputs its guess bit *c*' and wins if *c*' = *c*. A_ddh_ returns the output exactly as in the preceding experiment, except with (*X*, *Y*, *Z*) that was input to it. If A_ake_ outputs *c*, then A_ddh_ outputs 1; otherwise, it outputs 0. If (*X*, *Y*, *Z*) is a real extended chaotic map-based Diffie-Hellman triple, then A_ddh_ executes A_ake_ in ${\text{G}}_{\text{3}}^{ake}$ and so Prob. [event that A_ddh_ outputs 1] equals the Prob.[*E*_3_]. If (*X*, *Y*, *Z*) is a random triple, then A_ddh_ runs A_ake_ in ${\text{G}}_{\text{4}}^{ake}$ and so Prob. [event that A_ddh_ outputs 1] equals Prob.[*E*_4_]. Therefore,

$$|\text{Pr}[E_{\text{3}}]-\text{Pr}[E_{\text{4}}]|\leq Adv_{}^{ddh}({\text{A}}_{ddh}).$$ (5)

No information about flipping unbiased coin bit *c* is revealed, and all session keys are random and independent among all executions of the enhanced scheme. Thus,

$$\text{Pr}[E_{4}]=\frac{1}{2}.$$ (6)

Combining Eqs (1)–(6) and using Lemma 1, yields

$$Adv_{P}^{ake}({\text{A}}_{ake})\leq 2\cdot Adv^{ddh}({\text{A}}_{ddh})+\frac{2}{N}+\frac{1}{2^{l-1}}.$$

The proof is thus concluded.

#### Providing the contributory property of key agreements

Theorem 2. The enhanced scheme provides the contributory property of key agreements.

Proof: By Theorem 1, the session key security of the enhanced scheme is based on the extended chaotic map-based Diffie-Hellman problem. Therefore, the enhanced scheme avoids the security weakness that was proposed by Bergamo et al. [20] and neither a user nor the server alone can determine a session key. Thus, the enhanced scheme satisfies the contributory property of key agreements.

#### Withstanding replay attacks

Theorem 3. The password change phase of the enhanced scheme withstands replay attacks.

Proof: In the password change phase of the enhanced scheme, the smartcard sends the request message *M*_1_ = {*X*_1_,*X*_2_,*T*_1_} to the server, where *T*_1_ is the current timestamp, *X*_1_ = *T*_*a*_(*x*) mod *p*, *X*_2_ = *E*_*K*_(H* ∥ *Q* ∥ *R*), *K* = *T*_*a*_(*T*_*r*_(*x*)) mod *p*, H* = *h*(PW* ∥ *t*) and *Q* = *h*(*ID* ∥ *H* ∥ H* ∥ *T*_1_. By validating timestamp *T*_1_ and *Q* = ?*h*(*ID* ∥ *H* ∥ H* ∥ *T*_1_, the server can easily verify the freshness of the request messages that are received from the users, so the enhanced scheme withstands replay attacks.

#### Withstanding denial of service attacks

Theorem 4. The password change phase of the enhanced scheme withstands denial-of-service attacks.

Proof: Since the smartcard validates updated data R* by checking *Y*_*2*_ = *h*(*K* ∥ H* ∥ R* ∥ *T*_1_ and then replaces *R* with R*, where the timestamp *T*_1_ is generated by the smartcard and H* = *h*(PW* ∥ *t*), an attacker has difficulty in modifying the response message *M*_2_ = {*Y*_1_,*Y*_2_}. Therefore, the enhanced scheme withstands denial-of-service attacks.

#### Withstanding privileged insider attacks

Theorem 5. The password change phase of the enhanced scheme withstands privileged-insider attacks.

Proof: In the enhanced scheme, every legitimate user has (*x*,*T*_*r*_(*x*)) in his/her smartcard. By Theorem 1, the session key security of the enhanced scheme is based on the extended chaotic map-based Diffie-Hellman problem. Thus, a malicious user cannot derive the secret key *K* and the session key *λ* that is shared between another user and the server in the authenticated key exchange and the password change phases. Consequently, a malicious user cannot receive (*Q* ∥ *R*) and (*ID* ∥ *H* ∥ *CNT*) in the authenticated key exchange phase, and (H* ∥ *Q* ∥ *R*) and (*ID* ∥ *H* ∥ *CNT*) in the password change phases. Such a user has difficulty in forging valid request messages and impersonating other users. Thus, the enhanced scheme withstands privileged insider attacks.

### Logical analyses

This subsection describes the logical analyses of the proposed scheme by using the logical tool, which was defined and presented by Burrows et al. [46] in 1990 and Buttyan et al. [47] in 1998.

Assume that *P* and *Q* range over principals. *C* denotes a communicating channel and *X* and *Y* are messages. Table 1 defines the notation used for logical analyses [46–48].

**Table 1:** The notation used for logical analyses.

Symbol	Description	
*C*(*X*)	The message *X* is transited via channel *C*.	
*r*(*C*)	The set of readers of channel *C*.	
*w*(*C*)	The set of writers of channel *C*.	
*P*|≡ *X*	*P* believes the statement *X*.	
*P* |∼ *X*		*P* once said *X*.
*P*⊲*C*(*X*)		*P* sees *C*(*X*). The message *X* is transited via channel *C* and can be observed by *P*. *P* must be a reader of channel *C* to read message *X*.
*P*⊲ *X*|*C*		*P* sees *X* via *C*. The message *X* is transited via channel *C* and can be received by *P*.
(*X*)_*K*_		*X* is hashed with the key *K*.
$P\begin{array}{c} \underset{\leftarrow \to }{K} \end{array}Q$		*P* and *Q* can establish a secure communication channel by using the shared key *K*.

Table 2 lists the used assumptions and Table 3 lists the used logical description [46–48], where *A* and *B* are *S* and *U*, but *A* ≠ *B*.

**Table 2:** The assumptions of the proposed scheme.

(A1) *A* ∈ *r*(*C*_*A*,*B*_): *A* can read from the channel *C*_*A*,*B*_.
(A2) *A* ≡ (*w*(*C*_*A*,*B*_) = {*A*,*B*}): *A* believes that *A* and *B* can write on *C*_*A*,*B*_.
(A3) *A* ≡ (*B* ∥∼Φ → Φ): *A* believes that *B* only says what it believes.
(A4) *A* ≡# (*N*_*A*_): *A* believes that *N*_*A*_ is fresh.
(A5) $A\equiv \begin{array}{c} \underset{\to }{a}\\ \text{ECMDH(secret)} \end{array}A$: *A* believes that *a* is its extended chaotic map-based Diffie–Hellman secret.

**Table 3:** The inference rules of the logic of the proposed scheme.

Seeing rules
(S1) $\frac{P⊲C(X),P\in r(C)}{P\equiv (P⊲X|C),P⊲X}$: If *P* receives and reads *X* via *C*, then *P* believes that *X* has arrived on *C* and *P* sees *X*.
(S2) $\frac{P⊲(X,Y)}{P⊲X,P⊲Y}$: If *P* sees a hybrid message (*X*, *Y*), then *P* sees *X* and *Y* separately.
Interpretation rules
(I1) $\frac{P\equiv (w(C)=\{P,Q\})}{P\equiv (P⊲X|C)\to Q|\sim X}$: If *P* believes that *C* can only be written by *P* and *Q*, then *P* believes that if *P* receives *X* via *C*, then *Q* said *X*.
(I2) $\frac{P\equiv (Q|\sim (X,Y))}{P\equiv (Q|\sim X),P\equiv (Q|\sim Y)}$: If *P* believes that Q said a hybrid message (*X*, *Y*), then *P* believes that *Q* has said *X* and *Y* separately.
(I3) $\frac{P\equiv (\begin{array}{c} \underset{\to }{a}\\ \text{ECMDH(secret)} \end{array}P),P\equiv (\begin{array}{c} \underset{\to }{T_{b}(x)\text{mod}p}\\ \text{ECMDH(public)} \end{array}Q)}{P\equiv (P\begin{array}{c} \underset{\leftarrow \to }{T_{ab}(x)\text{mod}p} \end{array}Q)}$: If *P* believes that *a* is its extended chaotic map-based Diffie–Hellman secret and that *T*_*a*_(*x*) mod *p* is the extended chaotic map-based Diffie–Hellman component from *Q*, then *P* believes that *T*_*ab*_(*x*) mod *p* is the symmetric key shared between *P* and *Q*.
Freshness rules
(F1) $\frac{P\equiv (Q|\sim X),P\equiv \#(X)}{P\equiv (Q|\sim X)}$: If *P* believes that another *Q* said *X* and *P* also believes that *X* is fresh, then *P* believes that *Q* has recently said *X*.
(F2) $\frac{P\equiv \#(X)}{P\equiv \#(X,Y)}$: If *P* believes that a part of a mixed message *X* is fresh, then it believes that the whole message (*X*,*Y*) is fresh.
Rationality rules
(R1) $\frac{P\equiv (\Phi_{1}\to \Phi_{2}),P\equiv \Phi_{1}}{P\equiv \Phi_{2}}$: If *P* believes that Φ_1_ implies Φ_2_ and *P* believes that Φ_1_ is true, then *P* believes that Φ_2_ is true.

Then, according to [46–48], the proposed scheme is described in logic as follows.

$$\text{Step }1.\;S⊲(\begin{array}{c} \underset{\to }{T_{a}(x)\text{mod}p}\\ \text{ECMDH(public)} \end{array}U,C_{S,U}(h(ID||H||T_{1}),R),T_{1})$$

$$\text{Step }2.\;U⊲(\begin{array}{c} \underset{\to }{T_{b}(x)\text{mod}p}\\ \text{ECMDH(public)} \end{array}S,{\left(ID,h(ID||h(PW||t)||T_{1}),T_{2}\right)}_{\lambda },T_{2})$$

On the basis of to the assumptions and logical analyses, the proposed scheme must realize the following four goals of authentication and key agreement.

Goal 1: $U\equiv U\begin{array}{c} \underset{\leftarrow \to }{T_{ab}(x)\text{mod}p} \end{array}S$: User *U* believes that *λ* = *T*_*ab*_(*x*) mod *p* is a symmetric key shared between participants *U* and *S*.

Goal 2: *$S\equiv U\begin{array}{c} \underset{\leftarrow \to }{T_{ab}(x)\text{mod}p} \end{array}S$*: Server *S* believes that *λ* = *T*_*ab*_(*x*) mod *p* is a symmetric key shared between *U* and *S*.

Goal 3: *$U\equiv S\equiv U\begin{array}{c} \underset{\leftarrow \to }{T_{ab}(x)\text{mod}p} \end{array}S$*: User *U* believes that *S* is convinced of *λ* = *T*_*ab*_(*x*) mod *p* is a symmetric key shared between *U* and *S*.

Goal 4: *$S\equiv U\equiv U\begin{array}{c} \underset{\leftarrow \to }{T_{ab}(x)\text{mod}p} \end{array}S$*: Server *S* believes that *U* is convinced of *λ* = *T*_*ab*_(*x*) mod *p* is a symmetric key shared between *U* and *S*.

To accomplish the Goal 1, we have that

$$U\equiv \begin{array}{c} \underset{\to }{a}\\ \text{ECMDH(secret)} \end{array}U$$ (7)

and

$$U\equiv \begin{array}{c} \underset{\to }{T_{a}(x)\text{mod}p}\\ \text{ECMDH(public)} \end{array}U$$ (8)

must hold because of the interpretation rule (I3) and assumption (A5).

Next, to accomplish Eq (8), we have that

$$U\equiv (S||\sim (\begin{array}{c} \underset{\to }{T_{b}(x)\text{mod}p}\\ \text{ECMDH(public)} \end{array}S,{\left(ID,h(ID||H||T_{1}),T_{2}\right)}_{\lambda },T_{2})\to \begin{array}{c} \underset{\to }{T_{b}(x)\text{mod}p}\\ \text{ECMDH(public)} \end{array}S)$$ (9)

and

$$U\equiv (S||\sim \begin{array}{c} \underset{\to }{T_{b}(x)\text{mod}p}\\ \text{ECMDH(public)} \end{array}S)$$ (10)

must hold because of assumption (A3) and the rationality rule (R1). To accomplish Eq (10), we have that

$$U\equiv \#(\begin{array}{c} \underset{\to }{T_{b}(x)\text{mod}p}\\ \text{ECMDH(public)} \end{array}S)$$ (11)

must hold because of the freshness rules (F1), (F2) and assumption (A4).

To accomplish Eq (11), we have that

$$U\in r(C_{S,U}),$$ (12)

$$U\equiv (w(r(C_{S,U})=\{U,S\})$$ (13)

and

$$U\equiv ⊲C_{S,U}(\begin{array}{c} \underset{\to }{T_{b}(x)\text{mod}p}\\ \text{ECMDH(public)} \end{array}S)$$ (14)

must hold because of the interpretation rules (I1), the seeing rules (S1), (S2), assumptions (A1) and (A2). By using the interpretation rules (I3) and, we have the proposed scheme realizes

$$\text{Goal }1:U\equiv U\begin{array}{c} \underset{\leftarrow \to }{T_{ab}(x)\text{mod}p} \end{array}S.$$

Similarly, we have that the proposed scheme realizes Goal 2: *$S\equiv U\begin{array}{c} \underset{\leftarrow \to }{T_{ab}(x)\text{mod}p} \end{array}S$* by using the same arguments of Goal 1.

To accomplish Goal 3, we have that

$$U\equiv ((S||\sim U\begin{array}{c} \underset{\leftarrow \to }{T_{ab}(x)\text{mod}p} \end{array}S)\to (S\equiv U\begin{array}{c} \underset{\leftarrow \to }{T_{ab}(x)\text{mod}p} \end{array}S))$$ (15)

and

$$U\equiv (S||\sim U\begin{array}{c} \underset{\leftarrow \to }{T_{ab}(x)\text{mod}p} \end{array}S)$$ (16)

must hold because of the rationality rule (R1) and assumption (A3). To accomplish Eq (16), we have that

$$U\equiv (S|\sim U\begin{array}{c} \underset{\leftarrow \to }{T_{ab}(x)\text{mod}p} \end{array}S)$$ (17)

and

$$U\equiv \#(U\begin{array}{c} \underset{\leftarrow \to }{T_{ab}(x)\text{mod}p} \end{array}S)$$ (18)

must hold because of the freshness rules (F1), (F2) and assumption (A4). To accomplish Eq (18), we have that

$$U\in r(C_{U,S})$$ (19)

$$U\equiv (w(C_{U,S})=\{U,S\}),$$ (20)

and

$$U⊲C_{U,S}(U\begin{array}{c} \underset{\leftarrow \to }{T_{ab}(x)\text{mod}p} \end{array}S)$$ (21)

must hold because of the interpretation rule (I1), the assumptions (A1), (A2) and the seeing rules (S1) and (S2).

Thus, the proposed protocol realizes

$$\text{Goal }3:\;U\equiv S\equiv U\begin{array}{c} \underset{\leftarrow \to }{T_{ab}(x)\text{mod}p} \end{array}S.$$

Similarly, using the same arguments of Goal 3, the proposed scheme realizes Goal 4: *$S\equiv U\equiv U\begin{array}{c} \underset{\leftarrow \to }{T_{ab}(x)\text{mod}p} \end{array}S.$*

Therefore, the proposed scheme realizes Goals 1, 2, 3 and 4.

### Performance analysis and comparisons

Table 4 compares the performance and security properties of the enhanced scheme with related approaches [7, 9, 10, 15, 36, 37, 40, 49–53], where *T*_*H*_ denotes the time of executing a hash function operation; *T*_*C*_ denotes the time of executing a chaotic map operation; *T*_*S*_ denotes the time of executing a symmetric encryption/decryption operation; *T*_*SQ*_ denotes the time of executing a squaring operation; *T*_*SR*_ denotes the time of executing a squaring root solving operation; *T*_*M*_ denotes the time of executing a multiplication/division operation and *T*_*E*_ denotes the time of executing a modular exponential computation.

**Table 4:** Performance and security properties comparison.

Schemes	Computations	Transmissions		P_1_	P_2_	P_3_
Islam et al.’s scheme [15]	5*T*_*H*_+*T*_*SQ*_+*T*_*SR*_		2	Yes	Yes	Yes
Chen et al.’s scheme [9]	5*T*_*H*_+3*T*_*M*_+3*T*_*E*_		2	No	No	Yes
Jiang et al.’s scheme [10]	5*T*_*H*_+*T*_*M*_+5*T*_*E*_		2	No	Yes	Yes
Wang et al.’s scheme [49]	10*T*_*H*_		2	No	No	No
Lee et al.’s scheme [7]	16*T*_*H*_		3	No	No	No
Yan et al.’s scheme [50]	11*T*_*H*_		3	No	Yes	No
Das-Goswami’s scheme [51]	2*T*_*H*_+12*T*_*C*_		2	Yes	Yes	Yes
Lee et al.’s scheme [52]	12*T*_*H*_+4*T*_*C*_		2	No	No	Yes
He et al.’s scheme [53]	10*T*_*H*_+6*T*_*C*_		2	Yes	No	Yes
Islam et al.’s scheme [36]	18*T*_*H*_+10*T*_*C*_		2	Yes	No	Yes
Jiang et al.’s scheme [37]	21*T*_*H*_+6*T*_*C*_		2	Yes	Yes	Yes
Lin’s scheme [40]	5*T*_*H*_+5*T*_*S*_+6*T*_*C*_		2	Yes	No	Yes
Enhanced scheme	5*T*_*H*_+3*T*_*S*_+5*T*_*C*_		2	Yes	Yes	Yes

P_1_: Resisting possible attacks; P_2_: User anonymity; P_3_: Perfect forward secrecy.

The schemes proposed by Islam et al. [15], Chen et al. [9] and Jiang et al. [10] use the public key cryptosystem, require time-consuming modular exponential computations, and thus are inefficient. Although the schemes proposed by Wang *et al*. [49], Lee et al. [7] and Yan et al. [50] only employ the hash function operations and are more efficient than other schemes, these schemes fail to resist possible attacks and cannot provide perfect forward secrecy. The schemes proposed by Das and Goswami [51], Lee *et al*. [52], He et al. [53], Islam et al. [36], Jiang et al. [37] and Lin [40] and the enhanced scheme are based on chaotic maps and retain low computations and communications. Additionally, only the schemes proposed by Das and Goswami [51] and Jiang et al. [37] and the enhanced scheme resist potential attacks and provide more functions.

## Conclusions

This study addresses the weaknesses of Lin’s improved scheme including its vulnerability to denial-of-service attacks and privileged-insider attacks, and its inability to support the contributory property of key agreements. An enhanced smartcard-based password-authenticated key agreement scheme that is based on extended chaotic maps is presented. The session key security of the enhanced scheme is proven secure using the real-or-random and the sequence-of-game models, and it is based on the extended chaotic map-based DDHP. Thus, malicious users cannot derive a session key between another user and the server, and they cannot forge valid request messages or impersonate other users. Accordingly, the enhanced scheme withstands privileged insider attacks. Additionally, in the enhanced scheme, the messages that are sent from users are guaranteed to be fresh by the appending of timestamps, and the smartcard validates updated data from the server so the enhanced scheme withstands replay and denial-of-service attacks. Therefore, the enhanced scheme eliminates the weaknesses in previous schemes.
